# Exploring the Physical and Biological Aspects of BNCT with a Carboranylmethylbenzo[*b*]acridone Compound in U87 Glioblastoma Cells

**DOI:** 10.3390/ijms232314929

**Published:** 2022-11-29

**Authors:** Ana Belchior, Ana Fernandes, Maxime Lamotte, Andreia Filipa Ferreira da Silva, Raquel S. G. R. Seixas, Artur M. S. Silva, Fernanda Marques

**Affiliations:** 1 Centre for Nuclear Sciences and Technologies, Instituto Superior Técnico, Lisbon University, Nuclear and Technological Campus, Estrada Nacional 10, Km 139.7, 2695-066 Bobadela LRS, Portugal; 2 Department of Nuclear Sciences and Engineering, Instituto Superior Técnico, Lisbon University, Estrada Nacional 10, Km 139.7, 2695-066 Bobadela LRS, Portugal; 3 Department of Chemistry QOPNA, Aveiro University, 3810-193 Aveiro, Portugal

**Keywords:** boron neutron capture therapy, glioblastoma brain tumor, computational dosimetry, biological effects

## Abstract

Boron neutron capture therapy (BNCT) is a re-emerging technique for selectively killing tumor cells. Briefly, the mechanism can be described as follows: after the uptake of boron into cells, the thermal neutrons trigger the fission of the boron atoms, releasing the α-particles and recoiling lithium particles and high-energy photons that damage the cells. We performed a detailed study of the reactor dosimetry, cellular dose assessment, and radiobiological effects induced by BNCT in glioblastoma (GBM) cells. At maximum reactor power, neutron fluence rates were ϕ_0_ = 6.6 × 10^7^ cm^−2^ s^−1^ (thermal) and θ = 2.4 × 10^4^ cm^−2^ s^−1^ with a photon dose rate of 150 mGy·h^−1^. These values agreed with simulations to within 85% (thermal neutrons), 78% (epithermal neutrons), and 95% (photons), thereby validating the MCNPX model. The GEANT4 simulations, based on a realistic cell model and measured boron concentrations, showed that >95% of the dose in cells was due to the BNC reaction. Carboranylmethylbenzo[*b*]acridone (CMBA) is among the different proposed boron delivery agents that has shown promising properties due to its lower toxicity and important cellular uptake in U87 glioblastoma cells. In particular, the results obtained for CBMA reinforce radiobiological effects demonstrating that damage is mostly induced by the incorporated boron with negligible contribution from the culture medium and adjacent cells, evidencing extranuclear cell radiosensitivity.

## 1. Introduction

Glioblastoma multiforme (GBM) is one of the most malignant types of brain tumors in both adults and children. These tumors are normally associated with poor prognosis and a high level of morbidity and mortality across a wide range of patients. Despite substantial improvements in multimodal therapies, which include surgery, radiotherapy, and systemic therapy (chemotherapy, targeted therapy), glioblastoma remains a resilient cancer to the treatment options available [1,2]. 

A treatment plan for glioblastoma may combine several approaches. The first step is through surgical resection. However, in most cases, complete surgical removal of the entire tumor is extremely difficult due to its infiltration, extending into surrounding normal brain tissues [3]. Despite the substantial progress in the discovery of novel treatments, external beam radiotherapy with concomitant and adjuvant chemotherapeutic drugs (e.g., temozolomide) after surgery is the standard of care for GBM patients [4,5].

Targeted therapies that aim to selectively treat tumor cells while sparing normal cells and tissues have been proposed to improve the therapeutic ratio for traditionally difficult tumors like GBM. One of the strategies for selective destruction of the tumor and targeted delivery of chemotherapeutics without damaging the surrounding brain tissues is boron neutron capture therapy (BNCT) [6,7,8].

This emerging binary treatment modality is based on the nuclear capture and fission reactions that occur when the stable isotope boron-10 (^10^B) is irradiated with low-energy thermal neutrons (0.025 eV) (^10^B cross-section, 3848 barns), which leads to the production of an alpha particle and ^7^Li ions (^10^B + 1n → [^11^B]* → α + ^7^Li + 2.38 MeV). The short range of these alpha particles is typically a cell diameter (~ 10µm), which means that their energy deposition is limited to the diameter of a cell. Based on this quite attractive principle, it is possible to selectively irradiate cancer cells that have been pre-loaded with a sufficient amount of ^10^B, while sparing normal cells and tissues. Although BNCT has been considered an innovative form of radiotherapy with potential to treat many types of cancers, it remains an experimental treatment, dependent on the availability of innovative and specific drugs [9,10,11].

BNCT has characteristics that made this treatment modality promising for tumor treatment where surgery is ruled out (e.g., brain, head and neck). Nevertheless, there is still a need for better multi-disciplinary knowledge, more stress on clinical studies, and the availability of a reliable hospital-based neutron source [6].

The benefits are, among others, (i) good targeting and little damage to the surrounding healthy tissues; (ii) efficacy even for solid tumors affected by hypoxia; (iii) irreversible local cellular damage; (iv) use of an essential element (B) for human, animal, and plant health that can be combined with a variety of carriers; (v) possible advantage over other forms of particle therapy such as ions and protons due to the higher penetration of neutrons; (vi) lower dose deposited in healthy tissues along the neutron beam compared to protons; (vii) the low cost of neutrons [12].

The major challenge in BNCT is the selective accumulation in cancer cells of boron-containing agents and the delivery of therapeutic boron levels with minimal normal tissue toxicity. A number of candidate boron-containing agents for BNCT emerged decades ago, from simple boron-containing nucleic acids and peptides to boron-containing nanoplatforms [13]. Despite considerable effort, no new boron-containing drugs other than boronophenylalanine (BPA) and sodium borocaptate (BSH), which were synthesized and evaluated about sixty years ago and are considered 2nd-generation boron compounds, have FDA approval for clinical use thus far, although neither of these agents adequately fulfills the criteria of sufficient accumulation in the tumors and adequate selectivity [14,15]. 

More recently, with the advent of the 3rd-generation boron compounds, the focus has been to find compounds that work more specifically in targeting tumor cell components such as the nucleus and DNA to produce a more localized lethal effect [16].

An alternative to concentrate boron within the tumor cell nucleus is developing boronated compounds with DNA intercalators [17,18]. DNA intercalators such as acridine and acridone derivatives are excellent candidates, as they target the DNA and, at the same time, act as fluorescent probes to follow the compounds inside the cells [18]. In addition, these compounds have attracted considerable attention from the scientific community due to their wide range of biological activities, such as their broad spectrum of antitumor activities [19,20,21,22].

Benzo[*b*]-acridones have been much less studied than acridine/acridones. A biological evaluation of the potential of benzo[*b*]acridone derivatives bearing carboranyl moieties as BNCT agents was conducted by some of us in a previous publication [23]. The use of boron clusters instead of single boron atoms combine the high boron loading ability with high chemical and biological stability and low toxicity [24,25]. Although preliminary, results highlight the importance of these compounds as BNCT agents to treat GBM [23].

A safe and effective BNCT treatment depends on accurate calculation of the dose deposited in tumor. Therefore, the present work aimed to perform the dosimetry and microdosimetry of cell irradiations at the Portuguese Research Reactor (RPI) in the scope of ongoing research at CTN-IST for the development and in vitro evaluation of novel boron-based compounds for BNCT. We report the dosimetric characterization of irradiation facility at the vertical access of the RPI thermal column. Experimental methods for dose measurement and monitoring of cell irradiations were based on neutron foil activation and thermoluminescent photon dosimetry. The Monte Carlo particle transport simulation code MCNPX was used for a fine characterization of the mixed radiation field based on a detailed model of the irradiation facility coupled to an existing reactor core model. Simulations were extended to the evaluation of doses in cell structures using the Monte Carlo GEANT4 code, aiming at a correlation with the observed cell damage. The GEANT4 simulations, based on a realistic cell model and measured boron concentrations, show that >95% of the dose in cells was due to the BNC reaction. Correlation with the radiobiology studies demonstrated that damage was mostly induced by the incorporated boron, with negligible contribution from the culture medium and adjacent cells, evidencing extranuclear cell radiosensitivity. 

## 2. Results and Discussion

To estimate the absorbed dose involved in the cellular experiments and to correlate the cell damage with the dose delivered, we implemented a multidisciplinary study that included (i) Monte Carlo (MC) simulations using the codes MCNPX and GEANT, (ii) experimental measurements, and (iii) cellular studies. 

### 2.1. Charaterization of the Cellular Irradiation Facility

#### 2.1.1. Monte Carlo Simulations

**Figure 1:**
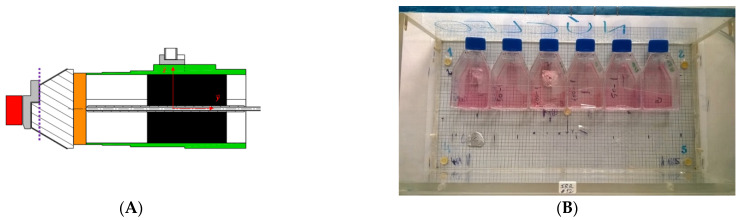
(**A**) Cut view of the thermal column. The core is represented in red. The source file of the core model was applied on the purple line. (**B**) The irradiation box used to expose the cells.

A set of simulations using the MC code MCNPX were performed to characterize irradiation facility. A detailed study of the radiation fields at the vertical access of the thermal column, where the cells were irradiated (see Figure 1A), was performed to interpret the foil measurements (by including the perturbation in the neutron field potentially induced by the samples) and address the contamination of the thermal neutron field by gamma rays and epithermal and fast neutrons. 

The calculated neutron energy spectrum in air shows a clear dominance of thermal neutrons (below 0.5 eV) over the total neutron population. Some fast neutrons are to be expected, as well as an epithermal component almost 10^4^ lower than the thermal neutron fluence. The calculated neutron fluence rates at 1 MW were ϕ_th_ = 7.59 × 10^7^ (thermal neutrons); θ = 1.96 × 10^4^ (epithermal neutrons) and ϕ_f_ = 4.82 × 10^4^ (fast neutrons). Next, the presence of the boron layer was investigated in terms of (i) perturbation induced by the samples on the radiation field and (ii) on spectrum temperature. There was a global increase of the neutron fluence rates up to 8.48 × 10^7^ (thermal), 2.06 × 10^4^ (epithermal), and 5.04 × 10^4^ (fast) due to the neutron reverberation in the hydrogen-containing materials. No significant change in the spectrum temperature appeared, despite the presence of the absorbing boron layer. For the angular distribution of the neutrons, most of them were transmitted across the surface of the cellular irradiation box (Figure 1B). Backscattering increased at low neutron energies, being responsible for the maxima observed for thermal and epithermal neutrons. Fast neutrons crossing the surface perpendicularly simply progressed without any further collision. The distribution of the neutron fluence rate along the *y* axis shows a clear decrease with the distance from the core. For fast neutrons, the evolution is approximately linear with y, while for thermal neutrons an inversely proportional distribution was observed. An average thermal neutron gradient of 5%cm^−1^ (from 10.51 to 5.59) × 10^7^ ncm^−2^ s^−1^ over 18 cm) was found. After the validation of the irradiation facility, we next investigated the neutron and photon doses in water. 

##### Neutron Doses in Water

The neutron dose was computed for a 1 mm water layer covering the entire irradiation box, which simulated the irradiated cellular monolayer. The simulated absorbed dose at the cellular monolayer was 8.50 ± 0.85 mGy·h^−1^.

**Figure 2:**
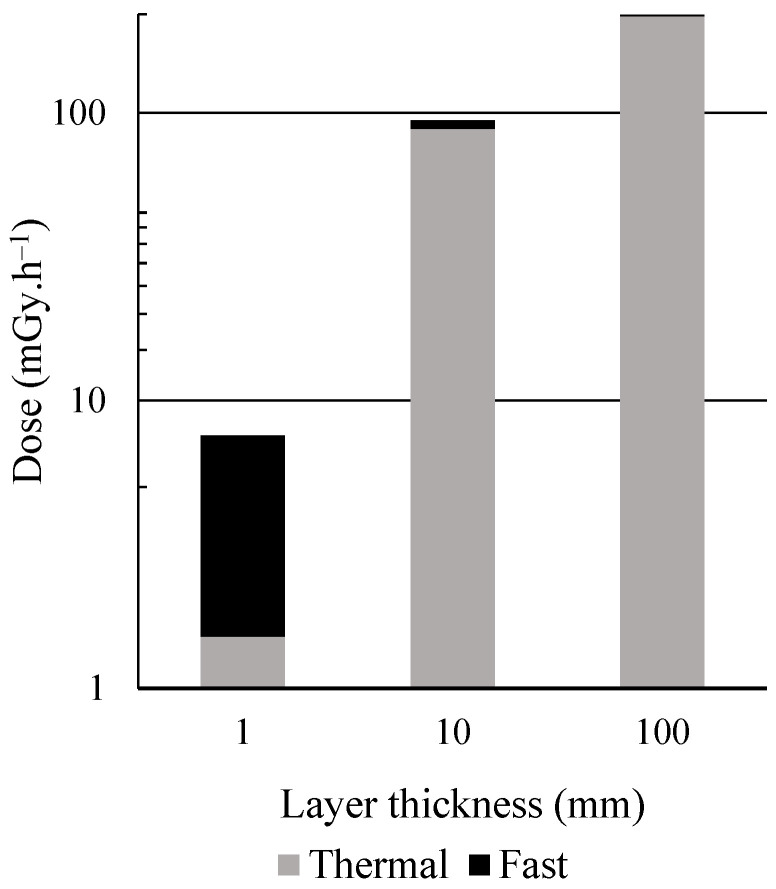
Dose induced by neutrons of different energy groups inside water layers of different thicknesses.

The influence of the sample thickness was also investigated for thermal neutrons. The released 2.2 MeV photons generated by (n,n’) reaction with water hydrogen travelled across the 1 mm layer without entirely depositing their energy: electrons above 1022 keV were observed as the main contributor to the dose. Fast neutrons mostly transferred their energy by elastic scattering, leading to high velocity protons and nucleus recoils that were stopped within the layer (thereby originating the high kerma factors at this energy range). As the volume increased, fast neutrons were thermalized and the gamma ray energies were totally absorbed; thermal neutrons were mainly responsible for dose delivery, as can be seen in Figure 2.

##### Photon Doses in Water

**Figure 3:**
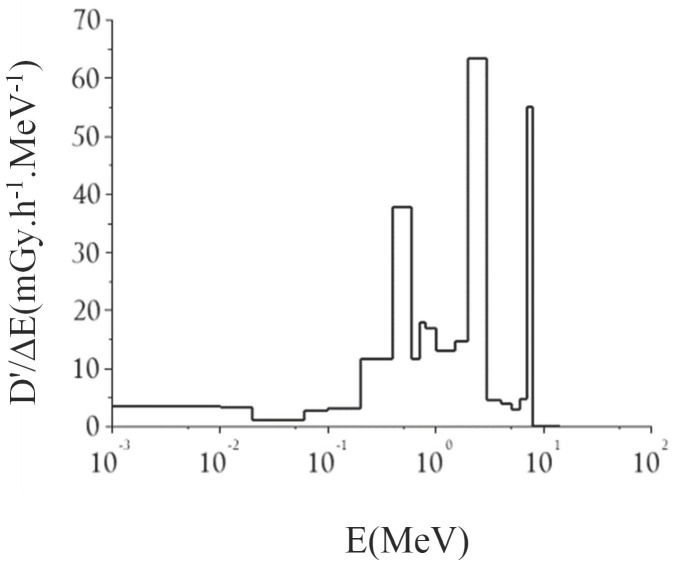
Photon–dose rate in water (in mGy·h^−1^MeV^−1^/MW) in the cellular irradiation box, normalized to 1 MW reactor power.

Regarding the photon dose in water, a value of 166 mGy·h^−1^/MW (±1.5%) was obtained from simulations in agreement with those obtained using TLD measurements, at 151 mGy·h^−1^/MW (Figure 3). Simulations of the cell samples yielded an increased photon dose of 253 mGy·h^−1^ (±1.3%) in air.

#### 2.1.2. Experimental Measurements

##### Neutron Fluence Rates

**Figure 4:**
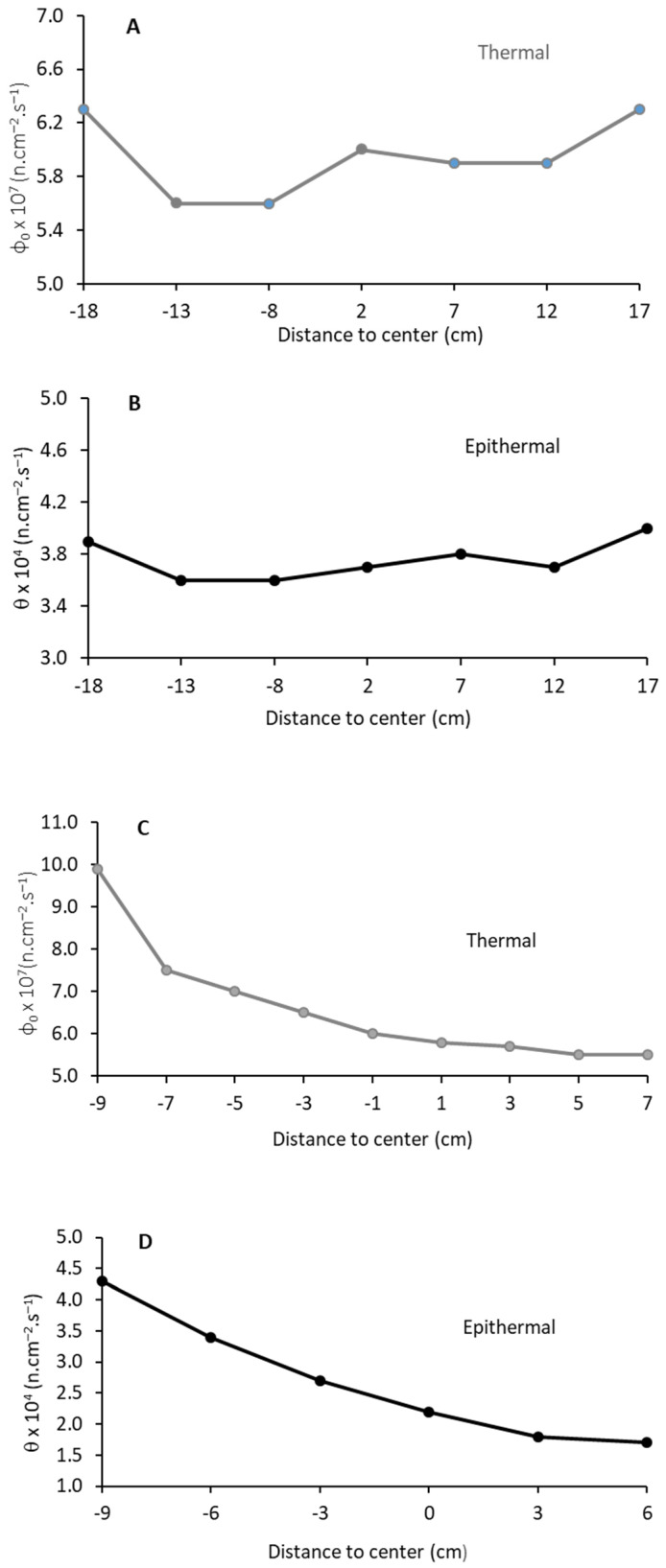
Neutron fluence rate distribution on the *x* axis (**A**,**B**) and *y* axis (**C**,**D**), normalized at 1 MW reactor power.

Thermal and epithermal neutron fluences were measured by the cadmium ratio method using two separate irradiations of bare and Cd-covered foils. The calculated self-shielding factors for the Au detectors were G_th_ = 0.96 (thermal) and G_epi_ = 0.28 (epithermal). For G_epi_, resonance data were extracted from ENDF B-VII.1. The neutron fluence distribution in the irradiation box was measured in the transversal and longitudinal directions. Seventeen bare Au foils were distributed in the irradiation box, forming a cross parallel to the 2 axes, and irradiated for 4 h. The distribution of epithermal neutron fluence employed 15 Cd-covered foils, irradiated for 8 h. The distributions of the thermal (ϕ) and epithermal (θ) neutron fluence rates are plotted with the distance to the center of the box in Figure 4A–D.

Table 1 displays the calculated and measured thermal fluence rates in the cellular irradiation box. The standard deviation of the simulations with different seed random numbers was in the order of 0.5% and is included in the uncertainty. The agreement between measurement and calculations is 15%. This value is generally acceptable considering an intrinsic accuracy of 10% for neutron transport simulations, the application of theoretical models describing neutron spectra, and the uncertainty in the self-shielding factors.

For epithermal neutrons, a steeper longitudinal distribution along the *y*-axis relative to thermal neutrons was found. The difference between calculated and measured epithermal fluence rate was 22% (Table 2). The measured thermal to epithermal fluence rate ratio was higher than calculated. This discrepancy indicates that an increased boron equivalent contamination should be considered due to the presence of other impurities in the graphite and/or lead. Chlorine is normally found in graphite (up to 30 ppm) due to the manufacturing process. Chlorine has a large absorption thermal cross-section and could further reduce the thermal neutron yield, In addition, the hygrometry of the graphite pile was not assessed and could over-moderate the fast neutrons increasing the epithermal component, and lead filters and shields may also contain several percent of antimony, tin, and other additives used to obtain better mechanical characteristics, leading to spectrum modifications.

##### Photon Dose Rate

**Figure 5:**
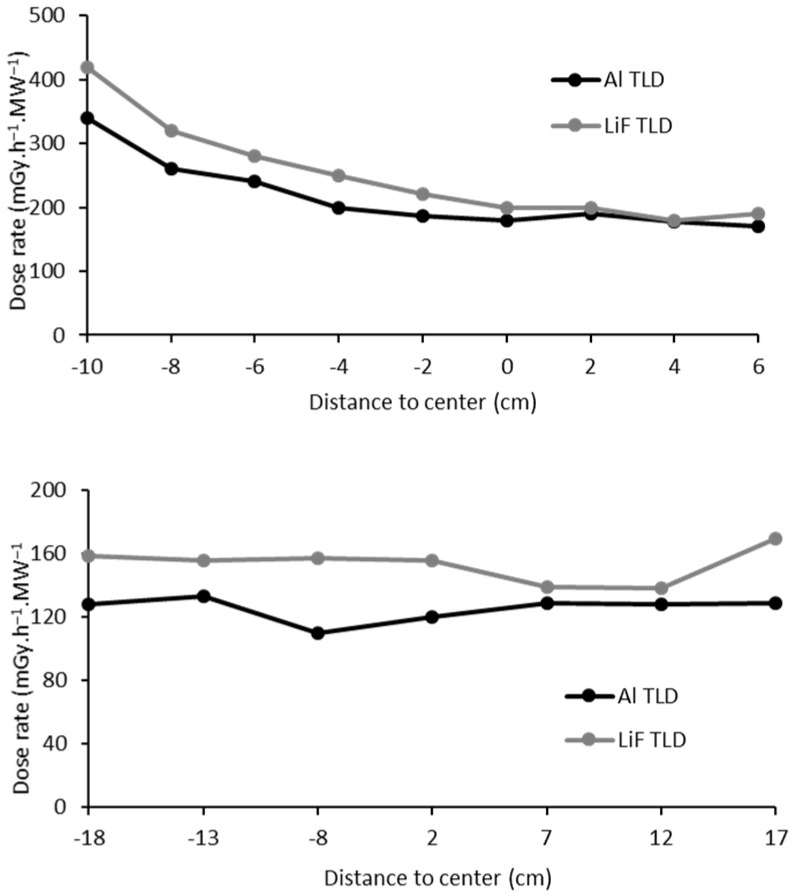
Photon dose profile on the *y*-axis (**up**) and *x*-axis (**down**).

As for neutrons, a higher dose rate was expected on the closest side to the reactor core (Figure 5). On the transversal axis x, no relevant fluctuation of the photon dose rate was observed, confirming the indivisibility of neutron and photon components.

TLD-700H yielded a systematically higher photon dose (by 14%) over TLD-500, which may be due to the application of nominal neutron sensitivity values. From this, an uncertainty of 10% was estimated for the photon dose measurements retrieved by the average results with both materials. The gamma dose in air showed an agreement of 95% between simulations and measures (Table 3) that is clearly smaller than the measurement uncertainty.

### 2.2. Assessment of Cellular Doses

About 10^5^ neutrons in each energy group (thermal, epithermal, and fast) were generated, in addition to 10^6^ photons, leading to a total of 462 interactions within the cell culture layer. Table 4 shows the contribution of the various radiations to the cell dose. The energy deposited by the incident photon component was negligible relative to the lithium and alpha recoils, similar to the epithermal and fast neutron contributions. The BNC reaction induced >95% of the total dose. If higher (>25 ppm) boron concentrations are encountered, the contribution of the BNC to the dose will be even larger, as fast and epithermal neutrons as well as photon reactions are independent to the boron amount in the culture.

The energy deposited by the incident photon component is negligible, similarly to the epithermal and fast neutron contributions. The BNC reaction induces >95% of the total dose. The dose rate at 1 MW reactor power is 602 mGyh^−1^ for an equivalent boron concentration of 25 ppm. This value is ~5 times larger than that retrieved by a simplified theoretical calculation. In order to understand this effect, a series of simulations were performed for considering B dispersed in an air layer (to avoid self-shielding and scattering from the medium), boron in a 1-mm-thick water layer in the culture flask with a (10 × 10) cm^2^ neutron source, and a similar set-up with a (50 × 50) cm^2^ source that embarked the lead shield and irradiation box. The results (Table 5) show that such an agreement was destroyed by the scattering in the flask and medium materials.

#### Dose in Cells

The simulation considered 10^6^ thermal neutrons incident on a layer of fifteen U87 cells (within a volume of 100 µm^3^). The doses received by the nucleus and cytoplasm of a single cell were calculated in conditions similar to the experiment. According to ICP-MS, there was a total cell uptake of 1187 ppm B, of which 15% was in the nucleus and 85% in the ensemble membrane + cytosol + cytoskeleton (Table 6). The dose received by the central cell was about 60 Gy for a thermal neutron flux of 4 × 10^12^ n·cm^−2^. No relevant difference was observed whether only the central cell or all cells were loaded with boron. In the extreme situation of a cell medium containing a high amount of boron (10,000 ppm), the dose in the cytoplasm increased by 0.8%. The dose in the cell is therefore induced mainly from the incorporated compound.

### 2.3. Cellular Studies

#### 2.3.1. Cellular Viability

**Figure 6:**
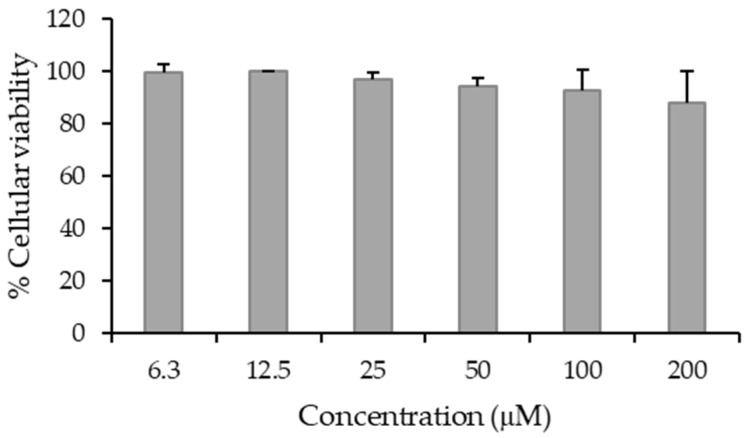
Cellular viability (%) of U87 cells upon 6 h treatment with CMBA at concentrations in the range of 6.3–200 µM. Results are expressed as a mean ± SD of two independent experiments with at least six replicates.

A low cytotoxicity for a BNCT agent is an important parameter so that boron concentrations within the tumor can be maximized with minimum toxicity. The aim of this experiment was to select a CMBA concentration for the irradiation experiments that by itself did not induce significant loss of cellular viability. As can be observed from Figure 6, CMBA did not show activity against the U87 cells at concentrations up to 200 µM.

#### 2.3.2. Cellular Uptake

**Figure 7:**
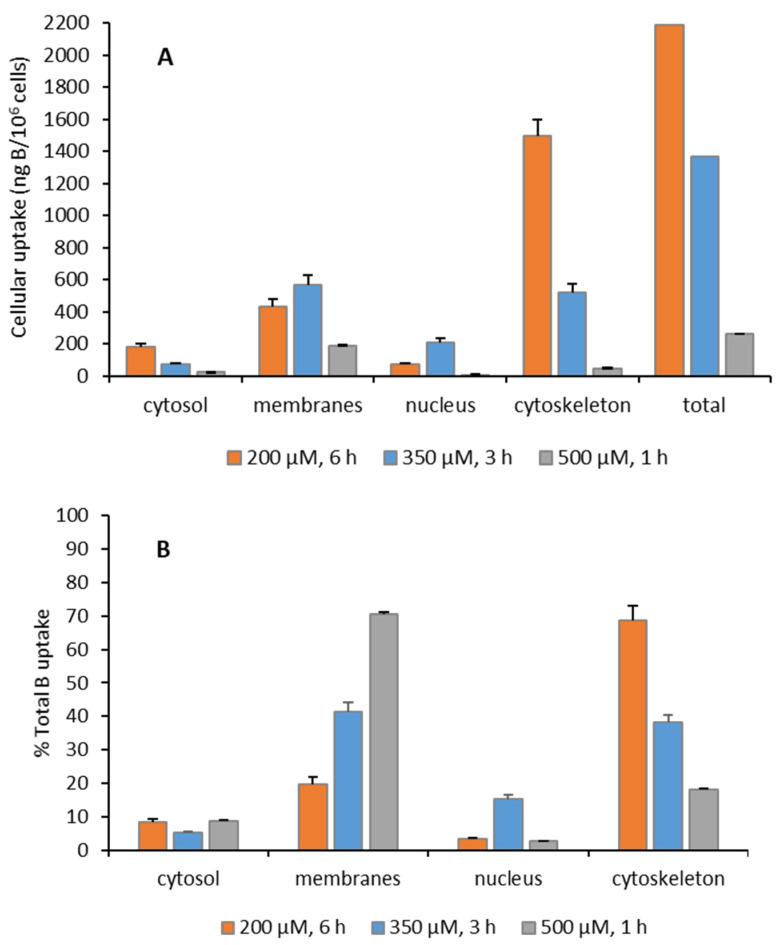
Cellular distribution of CMBA in the U87 cells. Cells were incubated with the compounds at 200, 350, and 500 μM upon 6, 3, and 1 h treatment, respectively. Results show the boron content found for each experimental condition in the cellular fractions. Boron levels are expressed as: (**A**) ng B/10^6^ cells and (**B**) % total B uptake.

The efficacy of BNCT depends on the boron concentration and boron distribution in the tumor cells. Therefore, the uptake of boron is crucial to obtain its distribution in tumor cells and even at the subcellular level. Inductively coupled plasma mass spectrometry (ICP-MS) is an analytical technique that allows very low detection limits (ppb levels) in comparison to other methods of analysis, with the advantage of also measuring different boron isotopes. The boron content was determined by ICP-MS in cytosol, membranes/particulate, nucleus and cytoskeletal fractions isolated from cells after exposure to CMBA. Figure 7 shows the comparative cellular distribution (Figure 7A) and total uptake (Figure 7B) at different concentrations: 200, 350, and 500 μM for 6, 3, and 1 h incubation with the U87 cells, respectively. Treatment of cells with the compound at different conditions showed that the total boron content was higher at 200 µM for 6 h incubation. Moreover, the cellular distribution profile differed for each condition—in particular, at 200 µM, where ca. 70% of total B was found in the cytoskeletal fraction. Results indicated that the concentration and time of incubation are important parameters for a better uptake. In addition, CMBA can target cellular components mainly at the membrane and cytoskeleton but without a cytotoxic effect, a main requisite for the design of new BNCT agents. Moreover, at 200 µM, the concentration of boron deposited into the cell is adequate for this therapeutic modality. We calculated an uptake of approx. 2.6 × 10^10 10^B atoms per cell, an amount superior to the target amount needed to yield lethal cellular damage following BNCT, which is ca. 1.2–2.1 × 10^9 10^B atoms per cell [26].

### 2.4. BNCT Study

#### 2.4.1. Cellular Viability after Irradiation

**Figure 8:**
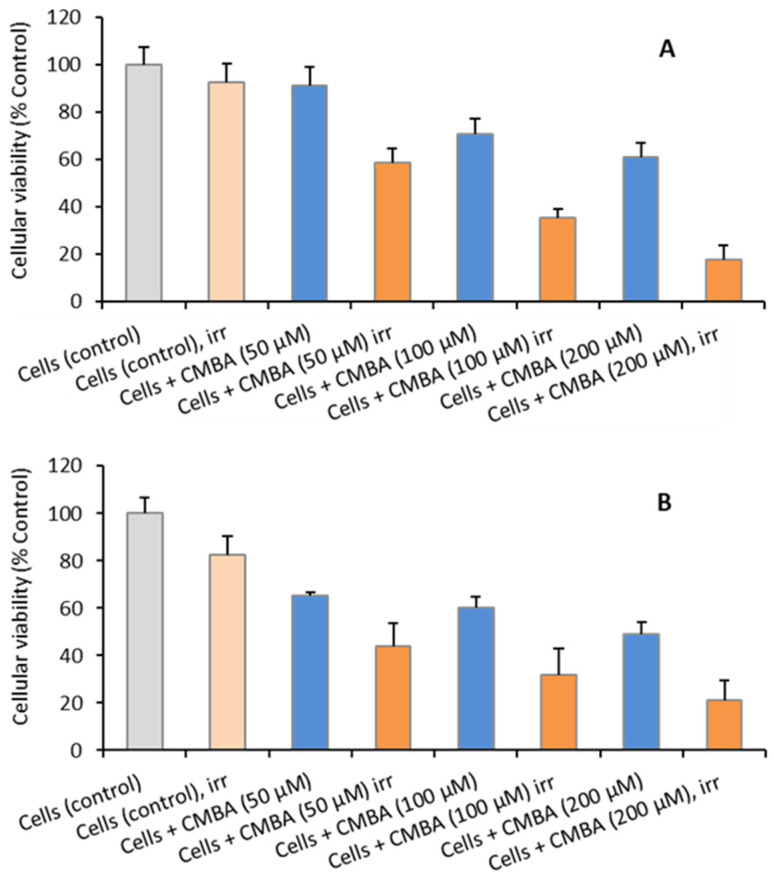
Cellular viability (%) of U87 cells after neutron irradiation (**A**) 24 h and (**B**) 72 h. Cells or cells previously incubated with CMBA at 50, 100, and 200 µM were irradiated at room temperature for 5 h at a nominal thermal neutron fluence rate of 6.6 × 10^7^ n cm^−2^ s^−1^, at 1 MW. Results are mean ± SD of 2 independent experiments with at least 4 replicates.

The general experimental procedure involved the irradiation of adherent U87 cells at ~70% confluence. Cell cultures irradiated in the thermal column of the research nuclear reactor included untreated cells (controls) and cells treated with CMBA at 50, 100, and 200 µM. The cellular viability of U87 cells are presented in Figure 8 after 24 h (Figure 8A) and 72 h post-irradiation (Figure 8B). 

#### 2.4.2. Biological Effects of Radiation

To determine the capability of CMBA to induce cellular lesions after neutron irradiation, we conducted the following studies: (i) the quantification of double-strand breaks (DSBs) using the ɣ-H2AX assay and (ii) the quantification of MN per BN cell using the cytokinesis-block micronucleus (CBMN) assay.

**Figure 9:**
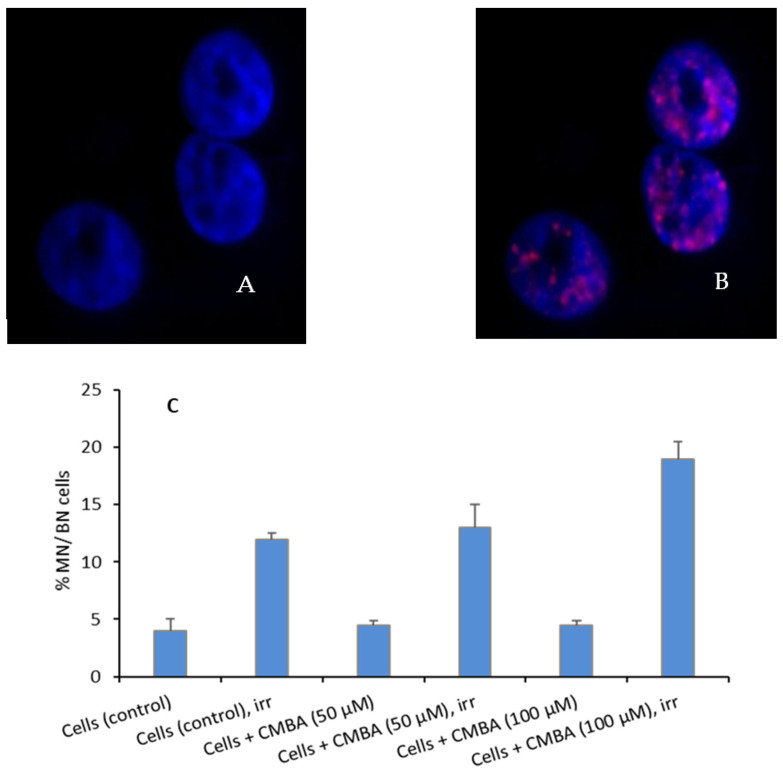
DSB quantification using the ɣ-H2AX assay (up); (**A**) represents the nuclei stained by DAPI, (**B**) the foci, pink dots, identified by TXR. The number of MN per BN cell is also presented for CMBA (down) (**C**). The error bars represent the standard deviation of the three independent experiments. The images were obtained using 64× magnification.

The ɣ-H2AX assay is a sensitive method for the early detection of double-strand breaks (DSB) in vitro and in vivo [27], and the CBMN assay assesses the chromosome damaging potential of different chemical and physical factors, including ionizing radiation-induced DNA damage [28]. MN is scored in binucleated cells, where cytokinesis is stopped by addition of cytochalasin B. The ability of CMBA to induce double-strand breaks (DSBs) was evaluated by a none quantitative preliminary test using the ɣ-H2AX assay, as shown in Figure 9A,B. Apart from the ability to induce DSBs, CMBA also induced an increased number of MN per BN cell in both concentrations (50 and 100 µM) post-irradiation when compared to control cells (*p* < 0.05) (Figure 9C). However, when compared with irradiated cells only, the number of MN per BN was not statistically increased (*p* = 0.48) for CMBA, with a concentration of 50 µM. Contrarily, the lesion induced by 100 µM post-irradiation was statistically increased (*p* < 0.05).

## 3. Materials and Methods

### 3.1. The Irradiation Facility

The thermal column contains a pyramidal stacking of graphite blocks inside the reactor pool, between the core and the reactor wall (thermal column extension), and an external pile. A lead shield of 24 cm thickness is placed between the core and the thermal column extension to shield against photons. The vertical access of the thermal column consists of a 1-m-diameter well for reaching the top surface of the external pile. Some graphite blocks were rearranged and a lead filter was added to form a cavity at the bottom of the well (2 m deep) that was very convenient for thermal neutron irradiation of monolayer cell cultures with a reduced photon background. At 1 MW, nominal fluence rates at the center of the cavity were ϕ_0_ = 6.6 × 10^7^ n·cm^−2^·s^−1^ and θ = 2.4 × 10^4^ n·cm^−2^·s^−1^ for thermal and epithermal neutrons, respectively. The radiation field was uniform along the transversal direction x, but the fluence rates decreased with the distance from the core (longitudinal direction y).

### 3.2. Monte Carlo Simulations

The MC code used was MCNPX version 6.1, developed and released by Los Alamos National Laboratory, Los Alamos, NM, USA [29]. This code facilitates fixed-source simulations (of various particle types) and neutronics studies. In this work, the existing core model was used to extract the source term for the subsequent simulation of the thermal column. The source term consisted of a file in which the positions and velocities of 40.7 million neutrons crossing a surface at 25 cm from the core were stored. The energy distributions (spectra) of neutron and photon fluences and photon energy fluence were calculated at the surface of the empty irradiation box; the target volume consisted of a 1-mm-thick air slab of 602 cm^2^ adjacent to the box surface. Dose calculations were based on flux-to-dose-rate conversion factors: kerma factors for neutrons and mass absorption coefficients for photons. Because the experiments dealt with biological samples, doses were calculated in air that represented the target volume and in water (the main tissue constituent). In the latter case, doses were also derived directly from the calculated energy deposited in the target volume filled with water. Finally, the number of neutrons crossing the surface of the irradiation box was simulated to extract their angular distribution. The energy bins for the F4 and F6 tallies were extracted from the BUGLE-47 group structure (47 neutrons and 20 photons). The first neutron energy bin (0–0.468 eV) includes the whole thermal region, and was therefore sub-divided in 38 bins to retrieve the detailed spectrum in this region. The angular distribution was retrieved in only 3 energy groups, corresponding to the thermal (<0.5 eV), epithermal (0.5–100 keV), and fast (>100 keV) regions. The target volume was divided into 4 equi-width transversal strips to obtain the longitudinal distribution of the neutron and photon fluences with respect to the distance from the reactor core.

#### Normalization and Coupling to Core Model

MCNPX outputs are relative to one source neutron. To scale the results to the emission rate of fission neutrons, a normalization factor *F* was applied:

(1) $$F=n_{f\;}\times \frac{\overset{=}{\upsilon }}{k_{eff}}\times P$$

where $\bar{\upsilon }$ is the average number of neutrons emitted per fission, *k_eff_* is the effective multiplication factor, and *P* is the reactor power in watt. The number of fissions per watt-second *n_f_* is

(2) $$\begin{array}{ll} n_{f}=\frac{1\;joule/sec}{\;watt} & \;\frac{1\;MeV}{1.602\;E-13\;joules}\;\frac{fission}{198\;MeV}\;\\ & \approx 3.15\;E\;10\;fission/watt-sec \end{array}$$

The values of $\bar{\upsilon }$ (2.42) and *k_eff_* (0.99) were extracted from the core model. The recoverable fission energy is known to depend mostly on the core materials (fuel, clad, coolant, reflector) and on the fuel burnup. For the fission of 235 U, values can vary from 198 to 207 MeV/fission [30]. The lower value was adopted, as it led to a good agreement between the calculated and measured neutron fluence rates using the RPI core model [31].

### 3.3. Neutron Fluence Rate Measurements

#### Activation Foils

**Figure 10:**
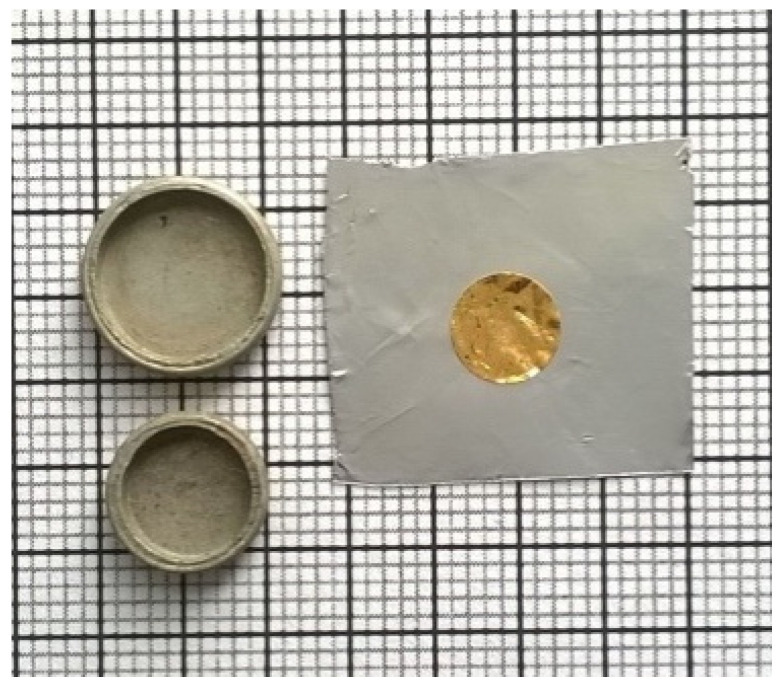
Gold detector and cadmium cover used for epithermal neutron fluence rate measurements.

The radiation field at the base of the irradiation box was characterized with gold foils for thermal and epithermal fluence rate measurements by the cadmium ratio method [31]. Circular foils of 100% Au with dimensions of Ø6 mm × 50 µm and weighing about 30 mg each were used (Figure 10). The activation reaction ^197^Au (n,γ)^198^Au was considered. Important data for this reaction include the following: a = 100.00%; M = 196.9665; T1/2 = 2.696 day; Eγ = 411.8044 keV; *p*γ = 95.56%; σ0 = 98.65b; I = 1550 b; g = 1.005 [32,33].

Foil activities were measured in a gamma spectroscopy system using a high-purity germanium crystal (HPGe) Camberra GC-2018. The system was calibrated for energy and efficiency using a set of standard pointwise photon sources of known activity. The efficiency depends on the crystal-to-source distance. A reference distance of 14.5 cm was used for calibration. Occasionally, it was necessary to attain higher efficiency by reducing the distance to 1 cm. The foil could no longer be considered pointwise, and an efficiency conversion was made by measuring at both distances a similar, activated Au foil with the same geometry. The uncertainty in each response measurement was estimated by error propagation, with a dominant contribution from the uncertainty in the source activity (1–2%). An overall uncertainty of 2% could also be derived from participation in a round-robin exercise on foil activity measurements [34].

### 3.4. Photon Dosimetry

Photon doses were measured with TLDs of Al_2_O_3_:C and ^7^LiF:Cu,P,Mg provided by Thermo Fisher Scientific under the designations of TLD-500 and TLD-700H, respectively. Dosimeter dimensions were ∅4.5 × 0.8 mm (TLD-500) and 3.2 × 3.2 × 0.3 mm (TLD-700H). These materials were selected for their low sensitivity to thermal neutrons. The measured relative thermal neutron sensitivity for TLD-500 ranged from 3.5 to 4.0. The thermal neutron sensitivity of TLD-700H has been reported as 3–10 smaller than that of ^7^LiF:Mg,Ti–a popular TLD similar to TLD-700H but with different dopants, for which 18 mGy ^60^Co/10^10^ n_th_cm^−2^ has been measured. The data are consistent with the application of a common value for the relative thermal neutron sensitivity of both materials, assumed as 3.5 mGy ^60^Co/10^10^ n_th_ cm^−2^. The TLDs were calibrated individually for kerma in air using a 60 Co source at the metrology laboratory of IST/CTN. The calibration was performed with 100 mGy, with a field size of (14.4 × 14.4) cm at 1 m from the source corresponding to an air kerma rate of 750 μGy·s^−1^ and 850 μGy·s^−1^ of dose in water. A set of 31 TLDs was simultaneously irradiated in an opaque Plexiglas support of 5 mm thickness.

### 3.5. GEANT4 Model

GEANT4 is a particle transport simulation code developed initially by CERN for high-energy physics. Its high-precision libraries allow for the simulation and analysis of the interactions generated by low-energy neutrons. The ability to track primary and secondary particles, as well as its high customization potential made it a good candidate for microdosimetric simulations and use in the GEANT4-DNA package for future DNA damage simulation.

**Figure 11:**
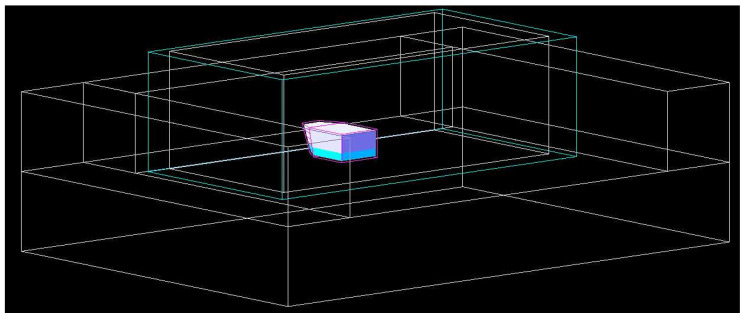
Geometry of the GEANT4 global geometry model for dose repartition.

The simulation aimed to quantify the collaboration of each kind of primary particle (neutrons and photons) and secondary particle (protons, alphas, electrons, photons from capture reactions, and scattered nuclei) to the overall energy deposited in the cell culture volume within the flask. The model comprised the irradiation cavity with the lead filter, irradiation box, culture flask, and chemical compounds (Figure 11).

The flask used for cell culture was made of polystyrene (1 mm thick) for its excellent optical characteristics, which are required for fluoroscopy analysis. The flask was placed along the longitudinal axis of the experience chamber at 2 cm from the wall of irradiation box closest to the reactor core. A single cell layer grew attached to the bottom of the flask. The cell layer was constructed as a 200 μm high by 25 cm^2^ (0.5 mL) volume. Based on the confluency of the cell culture from previous visual observations, the simulated cell layer is considered to be composed of 20% water and 80% brain cells: the same elemental composition as the brain according to ICRU reports. The concentration of natural boron was set to 25 ppm, a value corresponding to a CMA concentration of 230 µM. The homogenous liquid cell layer weighed 0.5132 g. Neutrons were generated in three energy groups: thermal, epithermal, and fast, each having the energy and angular distributions extracted from the MCNPX model. This discrimination helped in determining the interactions and deposited energy provoked by each energy group. For scaling, fast, epithermal, and thermal neutrons were assumed to compose, respectively, 0.11, 0.21, and 99.67% of the total neutron fluence rate in the thermal column, in accordance with MCNPX results and foil measurements. Photons were simulated with the MCNPX energy distribution and with isotropic emission. The particle emission surface was limited by the lead shield, with an overall area of 2500 cm^2^.

#### The Microscopic Model

**Figure 12:**
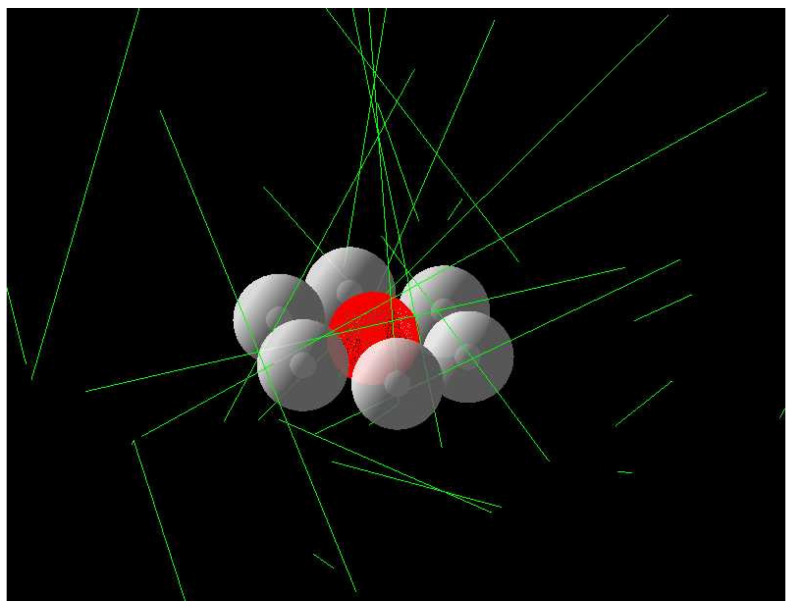
Geometry of the microdosimetric model; 25 neutrons were generated, some cells were hidden, and the central cell is sensitive.

The microdosimetric model of the cell cluster aimed to quantify the dose received by the U87 cells incubated with CMA at levels encountered during actual BNC experiments. Cells were represented in a planar cluster so that it was possible to monitor fragments from surrounding cells that may impact a central cell (cross-fire) (Figure 12). The central cell was composed of 2 structures: a cytosol shell and a nucleus sphere. The cytoskeleton, being geometrically located at the same place of the cytosol, was not represented, and its boron content was attached to the cytosol. The cell structures were assumed to have the same elemental composition as brain cells. The cell cluster contained 15 cells plus a central sensitive cell, where the doses were assessed. Three runs were conducted:

- Dose received by the central cell when all cells had boron;
- Dose received by the central cell when all but the central cells had boron;
- Dose received by the central cell if boron was present in surrounding water.

The model included an average cell uptake of 237 ppm ^10^B. This value corresponds to the upper limit obtained with a 350 μM concentration of CMA. During real irradiations, a lower concentration (50 to 200 μM) was used, presumably below detection limits of ICP-MS. This 237 ppm acted as an upper limit for dose estimation.

To decrease computation time, only thermal neutrons were generated from a planar source, as previous simulations showed that epithermal neutrons, fast neutrons, and photons had a very low impact on the overall dose.

### 3.6. Compound CMBA

**Figure 13:**
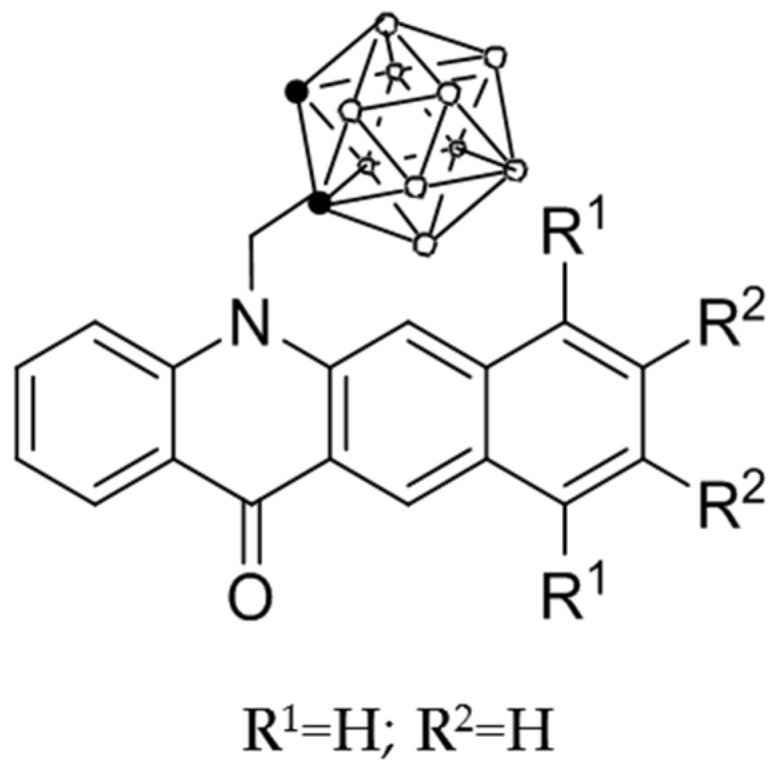
Schematic representation of 7-carboranylmethylbenzo[b]acridin-12(7H)-one, hereafter CMBA.

The compound 7-carboranylmethylbenzo[b]acridin-12(7H)-one, hereafter CMBA (Figure 13), was synthesized from 7-(propa-2-dien-1-yl)benzo[b]acridin-12(7H)-one and decaborane, as previously described [23].

### 3.7. Cell Culture

U87 MG (ATCC, HTB-14™) is a human malignant glioblastoma–astrocytoma cell line that is classified as grade IV. The cells were adherent epithelial cells and were cultured in DMEM (Dulbecco’s modified Eagle medium) + GlutamaxI (Gibco, Invitrogen) supplemented with 10% fetal bovine serum (Gibco) and 1% penicillin/streptomycin in a 5% CO_2_ incubator at 37 °C under humidified atmosphere.

#### 3.7.1. Cellular Viability Studies

The cellular viability after CMBA treatment either alone or after neutron irradiation was determined using the MTT assay based on the reduction of a yellow tetrazolium salt to purple formazan by metabolic active cells, as previously described [23]. For the selection of the concentration used in the irradiation studies, adherent cells (~10^4^ cells/200 µL) were incubated with CMBA in medium at serial concentrations in the range of 6.3–200 µM for 6 h at 37 °C, 5% CO_2_. Next, the medium was replaced by 200 μL of MTT (0.5 mg/mL PBS), and cells were incubated for a further 3 h. The water-insoluble formazan that was produced was then solubilized with 200 µL DMSO. The cellular viability was evaluated by measuring the absorbance at 570 nm in each well, which was taken as control (100% viable cells) for non-treated cells. A similar procedure was applied after neutron irradiation, i.e., 24 and 72 h post-irradiation.

#### 3.7.2. Cellular Uptake

U87 cells (~2 × 10^6^ cells/5 mL medium) were cultured in T25 flasks and incubated for 24 h to allow adherence. The medium was discarded, and CMBA was added to the cells. The compound was first diluted in DMSO and then in medium at different time points to 200, 350, and 500 μM. After 3 h of exposure at 37 °C, the solutions were removed, and cells were washed twice with cold PBS, detached with trypsin–EDTA solution (Gibco), and centrifuged to obtain a cellular pellet. The cytosol, membrane/particulate, cytoskeletal, and nuclear fractions were extracted using a FractionPREP™ cell fractionation system (BioVision, USA) according to the manufacturer’s protocol. The boron content in the different fractions was analyzed by a Thermo X-Series Quadrupole inductively coupled plasma mass spectrometer (ICP-MS) equipped with Ni cones and a glass concentric nebulizer (Meinhard, 1.0 mL min^−1^) refrigerated with a Peltier system. After digestion of the samples in 0.5 mL nitric acid (65%) (100 °C, 12 h) in a closed pressurized microwave digestion unit (Mars5, CEM) with a medium-pressure HP500 vessel, the samples were then diluted in ultrapure water to obtain 2.0% (*v*/*v*) nitric acid. The instrument was tuned using a multielement ICP-MS 71 C standard solution (Inorganic Venture). Indium (^115^In) at 10 µg/L was used as the internal standard.

#### 3.7.3. BNCT Studies: Cellular Irradiation

U87 cells were cultured in T25 culture flasks or in well plates according to the subsequent biological evaluation. For the radiobiological studies, cells (~10^6^/5 mL) were cultured in T25 culture flasks and incubated with CMBA at three different concentrations (50, 100, and 200 μM) 1 h prior to the irradiations. For the cytotoxic effect induced by neutron radiation, cells (approx. 10^4^) were cultured in 96-well plates and incubated with the compounds at 50, 100, and 200 μM. Then, the flasks or plates were placed into the thermal column of the Portuguese Research reactor (RPI), and cells were irradiated at room temperature for 5 h at 600 kW reactor power, corresponding to a maximum thermal neutron fluence of 5.8 × 10^11^ n cm^−2^ and photon dose imparted to the cells of 810 mGy.

#### 3.7.4. Cytokinesis-Blocked Micronucleus (CBMN) Assay

U87 cells were exposed to the irradiation procedure as described above. The number of micronuclei (MN) was determined by the cytokinesis blocked micronuclei assay. Briefly, 2 mg/mL cytochalasin-B was added to the medium 20 h after irradiation to arrest cytokinesis and cells were cultured for more 24 h. Then, cells were subjected to a hypotonic treatment (RPMI medium, water, and FBS), harvested, centrifuged (800 rpm, 10 min.), re-suspended twice in the wash solution (RPMI medium, water and FBS), and centrifuged again (800 rpm, 8 min.). The cells were fixed for 20 min. in cold methanol:acetic acid (3:1). Slides were prepared and stained in a solution of 4% Giemsa dye in phosphate buffer (pH 6.8) for 8 min. The slides were coded and scored under a light microscope (Zeiss, Portugal) at 40× magnification. MN were identified according to a previously described method [28]. The frequency of binucleated (BN) cells containing one or more MN was also scored.

#### 3.7.5. ɣ-H2AX Assay and Foci Analysis

U87 cells were washed three times with PBS and fixed with 4% formaldehyde in PBS for 15 min. After washing with PBS, cells were permeabilized with Triton X-100 (0.5%) at room temperature for 5 min., which was followed by two washing steps with 1% BSA in PBS. Then, cells were incubated with an anti-γ-H2AX primary antibody (mouse anti-γ-H2AX (ser139), Stressgen, bioreagents Corp., Canada) at 2 μg/mL for 1 h. After being washed twice with 1% BSA in PBS, cells were incubated with a FITC-conjugated anti-mouse secondary antibody (Santa Cruz Biotechnology, Santa Cruz, CA, USA) at 1 mg/mL for 1 h, followed by three washing steps with PBS. After incubation with Hoechst (Sigma-Aldrich, St. Louis, MO, USA) at 1 μg/mL for 5 min, cells were mounted in anti-fade mounting media (Vectashield H-100, Vector Laboratories, Burlingame, CA, USA).

## 4. Conclusions

A carboranylmethylbenzo[b]acridone compound (herein CMBA) was selected for this study due to its promising properties as a potential BNCT agent as previously reported. The major challenge in BNCT is that the boronated compound should accumulate into the cancer cells and deliver therapeutic boron levels with minimal toxicity, and CMBA fulfills all these requirements. Moreover, the significant accumulation in the cytoskeleton fraction was an important finding, considering that targeting the cell cytoskeleton is an important strategy in cancer therapy. In fact, cytoskeleton (actin and microtubule) play pivotal roles in cancer biology, as they regulate tumor relevant processes, such as cell cycle and cell proliferation.

This study used this compound to further explore radiobiological and physical aspects of the radiation field, especially the absorbed dose estimation and characterization, which constitute the most representative physical parameters that should be taking into account in BNCT.

A Monte Carlo model of the thermal column of the RPI was developed and employed to investigate the radiation field at this facility. A 10 ppm boron-equivalent contamination of the graphite was determined upon comparison with activation measurement. Simulated and measured outputs were within ±15, ±22, and ±5% for thermal neutrons, epithermal neutrons, and photons, respectively. This level of agreement is considered satisfactory in integral neutron fluence and photon dose measurements for reactor dosimetry. The Monte Carlo model of the experimental setup showed that the BNC reaction contributed more than 95% of cell doses, allowing for simplification of the irradiation dosimetry and microdosimetric model.

From microdosimetric simulations, the dose received by each cell with CMBA at 200 μM and exposed to a thermal neutron fluence of 8.20 × 10^11^ n_th_·cm^−2^ (corresponding to an irradiation at maximum reactor power for ~3 h) was 12 Gy.

The lack of agreement between microdosimetric Monte Carlo simulations and analytical calculations demonstrated the need of precise cell models for dose estimations.

The uptake of the compound by the nucleus did not seem to be critical, as double-strand breaks and late damage were observed upon BNC reaction.

To sum up, CMBA among the different proposed boron delivery agents showed promising properties due to its lower toxicity and important cellular uptake in U87 glioblastoma cells. BNCT dosimetry strongly depends on the parameters of the dose calculation models. Although further studies are needed in the context of radiobiology and dosimetric calculations, this work aimed to evaluate the dosimetry for in vitro experiments involving neutron irradiation of U87 monolayer cultured cells and assessing its future impact on the clinical settings for the treatment of glioblastoma.

## Figures and Tables

**Table 1:** Comparison of simulated and measured values for thermal neutron fluence rate.

	ϕ_th_ (n·cm^−2^ s^−1^)	ε (%)
Calculated	7.59 × 10^7^	4.31
Measured	6.56 × 10^7^	3.00

**Table 2:** Comparison of simulated and measured epithermal neutron fluence rate θ per unit lethargy and their respective relative uncertainties ℇ.

	θ (n·cm^−2^ s^−1^)	ε (%)
Calculated	1.96 × 10^4^	9.64
Measured	2.40 × 10^4^	3.00

**Table 3:** Comparison of simulated and measured values for photon dose rate D’_γ_ and their respective relative uncertainties ε.

	D’_γ_ (mGy·h^−1^)	ε (%)
Simulated	151.0	1.50
Measured	158.6	7.56

**Table 4:** Contribution of the different primaries to the overall deposited energy in the cell layer (Gy/n·cm^2^).

	^7^Li	Electrons	Alphas	Gammas	Protons	Nuclei	TOTAL	Contribution (%)
n_th_	7.70 × 10^13^	3.59 × 10^−15^	1.34 × 10^−12^	3.37 × 10^−16^	8.63 × 10^−21^	5.59 × 10^−22^	2.11 × 10^−12^	95.87
n_epi_	7.95 × 10^15^	8.80 × 10^−18^	1.39 × 10^−14^	2.51 × 10^−18^	4.38 × 10^−21^	5.14 × 10^−23^	2.19 × 10^−14^	0.99
n_fast_	1.71 × 10^−14^	1.27 × 10^−15^	2.01 × 10^−14^	0.01%	1.71 × 10^−14^	1.27 × 10^−15^	2.01 × 10^−14^	0.91
γ	0.00	4.90 × 10^−14^	0.00	5.88 × 10^−19^	0.00	0.00	4.90 × 10^−14^	2.22
							2.20 × 10^−12^	100

**Table 5:** Comparison of analytical and Monte Carlo dose in a boron volume.

	Analytical Dose (Gy/n·cm^2^/^nat^B ppm)	Simulated Dose (Gy/n·cm^2^/^nat^B ppm)	(Simulated/Analytical) Ratio
Boron in air	1.66 × 10^−14^	1.59 × 10^−14^	0.96
Boron in water, (10 × 10 cm source)	1.66 × 10^−14^	8.41 × 10^−14^	5.05
Boron in water, (50 × 50 cm source)	1.66 × 10^−14^	1.10 × 10^−13^	6.62

**Table 6:** GEANT4 simulation dose results of the microdosimetric model with respective relative uncertainty (ε) for 4 × 10^12^ n_th_.cm^−2^ (thermal neutrons).

	Cell Average		Cell Nucleus		Cell Cytosplasm + Cytosol	
Boron loading	D(Gy)	ε(%)	D(Gy)	ε(%)	D(Gy)	ε(%)
Central cell	59	5.98	36.8	11.95	58.7	5.72
All cells	60.5	5.75	35.7	12.22	56.4	5.78
